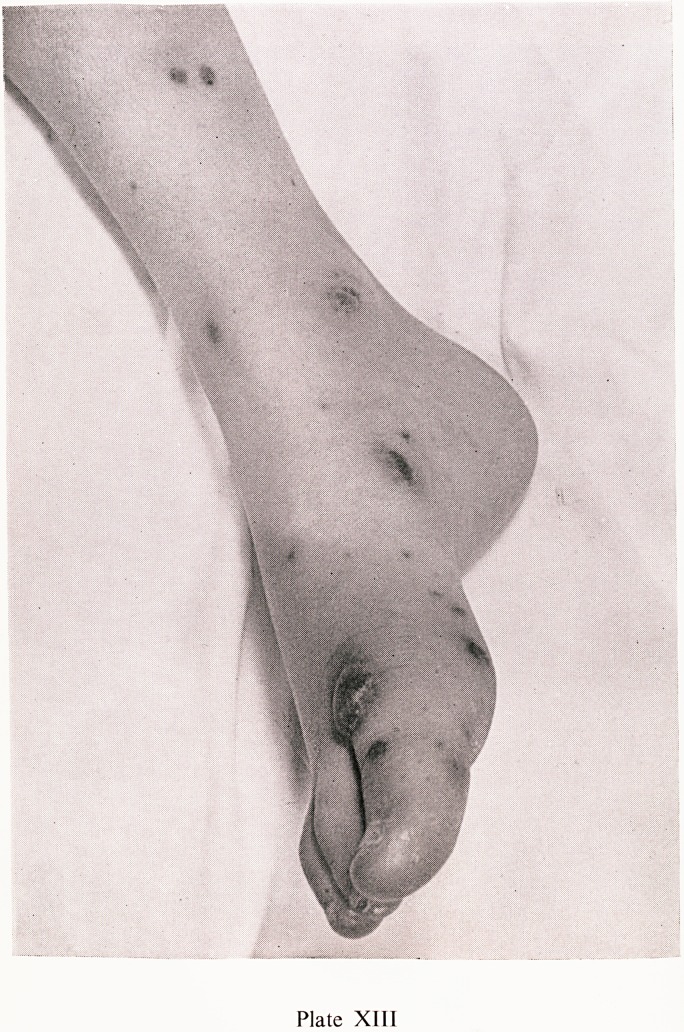# Anaphylactoid Purpura

**Published:** 1969-07

**Authors:** Margaret E. R. Stoneman


### ANAPHYLACTOID PURPURA

A Survey of the Patients Attending
The Bristol Royal Hospital for Sick Children
Between 1952 and 1967.

By

**Margaret E. R. Stoneman, M.D., M.R.C.P., D.C.H.**

Anaphylactoid purpura is a well-defined syndrome which is of fairly common occurrence in childhood. The characteristic rash and joint symptoms were first described by Schönlein in 1837, and Henoch in 1874 described the abdominal symptoms and recognised them as manifestations of the same syndrome. The most important feature of the syndrome, however, is the occurence in some cases of nephritis, which may occasionally progress to chronic renal failure. The present survey was undertaken in order to estimate the incidence of renal involvement in a large series of cases and to compare the clinical features of cases with and without renal involvement.

The diagnostic index of the Bristol Royal Hospital for Sick Children recorded 197 cases of anaphylactoid purpura between 1952 and the end of 1967 and it has been possible to trace and analyse the notes of 191 of these, of whom 62 had evidence of renal involvement. One of the children whose notes could not be traced is known to have died from renal failure in 1962 and inclusion of this case brings the incidence of renal involvement to 63 out of 192 cases (33 per cent).

The 191 children whose records were available are now considered in detail and compared with those in previously reported series.

### AETIOLOGICAL FACTORS

The aetiological factors in this and other published series are summarised in Table I.

**Sex.** The series included 115 males (60 per cent of the total) and 76 females. The increased incidence in males has been noted in most of the previously reported series, with the exception of that of Bywaters *et al.* (1967). In the present series there was a similar sex ratio in cases with and without renal involvement.

**Age.** The age incidence of the children in this series is similar to that in previously reported series and is shown in Fig. 1. The peak is from three to seven years. The disease is rare before the age of two years, and the incidence falls steeply at nine years. It will be seen, however, that the incidence of renal involvement is higher and its duration frequently longer in the older children. This is similar to the findings of Wedgwood and Klaus (1955), who found in a long-term follow-up that 69 per cent of children who were over six years of age at the onset had abnormal urines as compared with 8 per cent of the younger children.

**Seasonal Incidence.** The seasonal incidence of the cases in this series is shown in Fig. 2. It was higher in the autumn and winter months, as was reported by previous authors (Lewis, 1955; Derham and Rogerson, 1956; Bywaters

**TABLE I. Aetiological Factors in Anaphylactoid Purpura**

| Author | Number of Cases | % Males | Age | History of Infection | Haem. Strep. + | A.S.O. 7200 | History or Evidence of Infection |
|---|---|---|---|---|---|---|---|
| Gairdner (1948) | 12 | 83% | 4—15 years (+2 adults) | 42% | 92% | | |
| Balf (1951) | 20 | 75% | 2—11 years (Max. 4—8) | | | | |
| Derham & Rogerson (1952) | 35 | | | 54% | 71% | | |
| ,, ,, (1956) | 59 | | | 25% | 36% | | |
| ,, ,, (Total) | 94 | 54% | 1-8/12—14 yrs. (Max. 5—7) | | | | |
| Philpott (1952) | 40 | 63% | months—10 yrs. (Mean 5.2) | 50% | | | 55% |
| Lewis (1955) | 139 | 58% | 35% pre-school) (Max. 4—5) | 50% | 24% | | |
| Wedgwood & Klaus (1955) | 26 | 65% | 1-1/12—12½ yrs. (Max. 2—4) | 50% | 56% | | |
| Oliver & Barnett (1955) | 26 | 65% | 11 mths.—10 yrs. (Mean 4.3) | | | | |
| Rubin (1955) | 65 | | | | 70% | | |
| Bywaters *et al* (1957) | 64 | 50% | Max. 5—7 yrs. | 72% | 23% | 22% | |
| **Bristol Series** | | | | | | | |
| No renal involvement | 129 | 60% | See Fig. 1 | 74% | 30% | 38% | 85% |
| Renal involvement | 62 | 61% | | 78% | 24% | 47% | 84% |
| Total | 191 | 60% | | 75% | 28% | 41% | 85% |

*et al.* 1957) There was much less variation in seasonal incidence in those cases with renal involvement, and similar findings were reported by Lewis (1955) in 297 cases of acute nephritis.

FIG. 1. AGE INCIDENCE IN 191 CASES OF ANAPHYLACTOID PURPURA.

**History of Infection.** Anaphylactoid purpura is considered to be a manifestation of hypersensitivy, the sensitising agent in the majority of cases being an infection, commonly with a beta-haemolytic streptococcus. In the present series a history of preceding infection was obtained in 143 cases (75 per cent), an incidence similar to that reported by Bywaters *et al.* (1957), and higher than that in earlier series. Evidence of haemolytic streptococcal infection was found in 71 (37 per cent), as shown by isolation of the organism from throat swabs and/or an antistreptolysin-O titre of 200 or more; and in one case

FIG. 2 SEASONAL INCIDENCE IN 191 CASES OF ANAPHYLACTOID PURPURA

▒ = Cases with renal involvement.

■ = " " " " of over one month's duration.

there had been a preceding attack of scarlet fever. A further 35 patients had had treatment with antibiotics before attending hospital. Only 29 patients

Plate VIII

Plate IX

Plate X

Plate XI

Plate XII

Plate XIII

(15 per cent) had neither a history of infection nor laboratory evidence of haemolytic streptococcal infection. There was no difference in the incidence of preceding infection in those cases with and without renal involvement.

### PATHOLOGY

The histological characteristics of the skin lesions were described by Gairdner (1948). He found an aseptic inflammatory reaction affecting the small vessels of the corium, sometimes accompanied by haemorrhage and in severe cases proceeding to a necrotising arteriolitis. He considered that the gastro-intestinal, articular, and renal lesions, could all be explained by similar vascular lesions.

### CLINICAL FEATURES

**Onset.** The onset usually occurred from one to two weeks after a preceding upper respiratory tract infection. Joint symptoms alone were the presenting feature in 50 cases (26 per cent), the rash alone in 46 cases (24 per cent), and rash and joint symptoms occurred simultaneously in 38 (20 per cent). Abdominal symptoms were the presenting feature in 39 cases (20 per cent) and nephritis in four cases ( 2 per cent). The remaining 14 patients presented with mixed signs and symptoms.

The incidence of signs and symptoms in this and previously published series is summarised in Table II.

**Rash.** The rash, which commences as urticarial papules, flattening to macules and becoming purpuric later, is present in almost every case. The characteristic distribution is shown in Plates VIII, IX and X, the buttocks and extensor surfaces of the limbs being predominantly affected. In very young children the rash may also occur on the face, as in the nine month old child shown in Plates XI and XII. In severe attacks the skin lesions may be necrotic, as seen in Plate XIII.

**Joint Symptoms.** Joint pain, frequently accompanied by transient swelling, affects mainly the knees and ankles, and occasionally hips, wrists or elbows. If joint involvement precedes the rash, rheumatic fever may be suspected, but in most cares the rash appears soon after or simultaneously with the joint symptoms. In the present series 81 per cent of children had joint symptoms, a similar incidence to that reported by Gairdner (1948), and higher than in other series. There was no difference in the incidence of joint symptoms in patients with and without renal involvement.

**Abdominal Symptoms.** The incidence of abdominal symptoms has varied considerably in the reported series (See Table II). In the present series abdominal pain was present in 67 per cent of cases, vomiting in 59 per cent, and gastro-intestinal bleeding, either visible or occult, in 46 per cent. 78 per cent of patients had one or more of these abdominal symptoms, and they were the presenting feature in 20 per cent. One patient had an operation for intussusception a month before the appearance of the rash, and the appearance of the appendix, which was involved in the intussusception, was typical of the intestinal lesions described in anayphylactoid purpura (Balf, 1951). In three other cases the abdominal symptoms led to a laparotomy being performed. The incidence of abdominal symptoms was higher (95 per cent) in the presence of renal involvement than in its absence (70 per cent).

**TABLE II. Incidence of Signs and Symptoms in Anaphylactoid Purpura**

| Author | Number of Cases | Rash | Joint Symptoms | Abdominal Pain | Vomiting | Gastro-Intestinal Bleeding | One or more Abdominal Symptoms | Nephritis |
|---|---|---|---|---|---|---|---|---|
| Gairdner (1948) | 12 | 100% | 83% | 92% | 50% | 67% | | 92% |
| Balf (1951) | 20 | 100% | 60% | 80% | | 25% | | 50% |
| Derham & Rogerson (1962) | 35 | 100% | 57% | 74% | | 31% | | 54% |
| ,, ,, (1956) | 59 | 100% | 59% | 51% | 37% | 31% | | 46% |
| ,, ,, Total) | 94 | | | | | | | 49.5% |
| Philpott (1952) | 40 | 100% | | | | | | 47.5% |
| Wedgwood & Klaus (1955) | 26 | 100% | 62% | 42% | 27% | 19% | | 50% |
| Oliver & Barnett (1955) | 26 | | | | | | | 42% |
| Diamond (1955) | 75 | | | | | | | 44% |
| Rubin (1955 ) | 65 | | | | | | | 20% |
| Bywaters *et al* (1957) | 64 | 98% | 67% | 58% | | 28% | | 40% |
| **Bristol Series** | | | | | | | | |
| No renal involvement | 129 | 99% | 81% | 60% | 49% | 38% | 70% | |
| Renal involvement | 62 | 98% | 79% | 82% | 79% | 61% | 95% | |
| Total | 191 | 99% | 81% | 67% | 59% | 46% | 78% | 32% |

**TABLE V. Incidence of Persistent Renal Abnormality in Anaphylactoid Purpura.**

| Author | Total Cases | Cases with Renal Involvement | Fate Unknown | Complete Recovery | Persistent Abnormality: Died with Renal Failure | Chronic Nephritis | Latent Nephritis | Total with persistent renal abnormality: Number of Cases | % of cases with renal involvement | % of total cases with purpura |
|---|---|---|---|---|---|---|---|---|---|---|
| Gairdner (1948) | 12 | 11 | | 6 | 1 | 2 | 2 | 5 | 45% | 42% |
| Philpott (1952) | 40 | 19 | 8 | 7 | | | 4 | 4 | 36% | 10% |
| Wedgwood &Klaus (1955) | 26 | 13 | | 3 | | | 10 | 10 | 77% | 39% |
| Oliver & Barnett (1955) | 26 | 11 | | 8 | | | 3 | 3 | 27% | 12% |
| Derham & Rogerson (1956) | 94 | 51 | | 40 | 2 | 4 | 5 | 11 | 22% | 12% |
| Bywaters *et al* (1957) | 64 | | | | | | | | | 10% |
| **Bristol Series** (if 12 cases whose fate unknown all failed to clear) | 192 | 63 | 12 | 48 | 2 | 1 | | 3 (15) | 5% (24%) | 2% (8%) |

**Nephritis.** Evidence of renal involvement, as shown by the presence of albumen or red cells in the urine, was present in 32 of the cases in the present series, a lower incidence than that found in all other series with the exception of that of Rubin (1955). In four cases nephritis preceded other signs by periods of from three to nine months.

The present series has been divided into 27 patients in whom the signs of renal involvement cleared within one month, and 33 in whom they persisted for more than one month. In two cases, followed up at hospitals in other areas, the duration of nephritis is not known.

**TABLE III. Time of Onset of Nephritis Related to its Duration**

| Time of Onset of Nephritis | Duration of Nephritis Less than 1 month | Duration of Nephritis Over 1 month |
|---|---|---|
| Less than 1 week | 8 | 6 |
| 1—2 weeks | 12 | 3 |
| (Within 2 weeks) | (20) | (9) |
| 2—3 weeks | 0 | 3 |
| 3—4 weeks | 1 | 7 |
| 1—2 months | 3 | 3 |
| 2—3 months | 1 | 4 |
| 3—6 months | 1 | 0 |
| 6—12 months | 0 | 0 |
| Over 12 months | 0 | 2 |
| (After 2 weeks) | (6) | (19) |
| Not known | 0 | 2 |
| Before Other Symptoms | 1 | 3 |
| Total | 27 | 33 |

The time from the onset of signs and symptoms of anaphylactoid purpura to the first evidence of renal involvement is shown in Table III. It will be seen that in 29 cases in which evidence of renal involvement appeared within two weeks, the duration of nephritis was less than one month in 20 (69 per cent), whereas in 25 cases with a later onset, the nephritis lasted for more than one month in 19 (76 per cent). The duration of signs of renal involvement in those cases in which they eventually disappeared is shown in Table IV. It will be seen that signs cleared completely in 48 cases (76 per cent of those with renal involvement). Of the remaining children, the fate of twelve whose urine was abnormal when last examined is unknown, ten having failed to keep follow-up appointments and two living outside the area. One child, who is still attending hospital $4\frac{1}{2}$ years from the onset of symptoms, has chronic nephritis with hypertension. One child died from renal failure, and another child with Down's syndrome and a congenital heart lesion still had an abnormal urine at the time of death from cardiac failure, three years after the onset of the purpura.

**TABLE IV. Duration of Renal Involvement in 48 Cases in which resolution was observed.**

| Duration of Renal Involvement | Number of Cases |
|---|---|
| Less than 1 week | 15 |
| 1—2 weeks | 5 |
| 2—3 weeks | 2 |
| 3—4 weeks. | 5 |
| Total : Less than 1 month | 27 |
| 1—2 months | 2 |
| 2—3 months | 5 |
| 3—6 months | 5 |
| 6—9 months | 2 |
| 9—12 months | 1 |
| Over 12 monthhs | 2 |
| Duration unknown (but known to have cleared later) | 3 |
| Duration over 1 month, cleared later | 21 |
| No evidence of renal involvement when last seen | 48 |

The incidence of known permanent renal impairment is therefore 5 per cent of the total with nephritis, or 2 per cent of the total cases of anaphylactoid purpura. If the twelve patients whose fate is unknown all had permanent renal impairment, which is unlikely, the total incidence of permanent renal impairment would be 24 per cent of the total with nephritis, or 8 per cent of the total cases of anaphylactoid purpura. Even these hypothetical maximum figures are lower than those reported by Wedgwood and Klaus (1955) who carried out Addis counts in 26 children who had had anaphylactoid purpura and found abnormal urines in 10 (39 per cent), and by Gairdner (1948) who reported one death from uraemia, two cases of chronic nephritis and two of latent nephritis in his twelve cases, an incidence of persistent renal abnormality of 42 per cent. The incidence in the present series is more in keeping with findings of Philpott (1952), Oliver and Barnett (1955), Derham and Rogerson (1956) and Bywaters *et al.* (1957) who all found that between 10 per cent and 12 per cent of their cases of anaphytactoid purpura had persistent renal abnormality. These published reports are summarised in Table V.

Analysis of the cases in the present series shows that renal involvement is more likely to occur in older children and in those with abdominal symptoms, and is more likely to persist when the onset of nephritis is delayed until after the second week of the illness.

**Other Complications.** Febrile convulsions occurred in five children and meningism in one. One child had an operation for intussusception a month before the appearance of other symptoms, and three others had a laparotomy for abdominal symptoms before the appearance of the rash. One child had an operation for a perforated ileum nine months after the onset. The appearances at laparotomy in this case were said to be typical of those seen in perforation due to a foreign body, although no foreign body was found. Two children with the nephrotic syndrome also had other complications, one developing paralytic ileus and one anorexia nervosa. One child had transient thrombocytopenia.

**Associated Conditions.** Two children had infective hepatitis, one had Down's syndrome with a congenital heart lesion, and there was one case each of asthma, coeliac syndrome, and tuberculous cervical adenitis.

**Conditions Developing Later.** Two children subsequently suffered from epilepsy, one developed diabetes mellitus $2\frac{1}{2}$ years later, one had rheumatic fever 7 years later and one had a cerebellar tumour removed 12 years later.

**Duration of Illness.** In many cases the illness was mild and 38 (20 per cent) were not admitted to hospital. In 111 cases (58 per cent) the stay in hospital was less than one month and in 29 cases (15 per cent) it was between one two months. Only 13 children (7 per cent) were in hospital for more than two months.

Recurrences of rash and joint symptoms not severe enough to necessitate re-admission to hospital were fairly common. Twelve children required a further admission to hospital for relapse or recurrence of symptoms, seven within three months of the onset and two of these had a third admission. One child who was not admitted in the first attack required admission in a second attack over a year later.

Fifteen (45 per cent) of the children who had renal involvement lasting for more than one month had had a relapse or recurrence of symptoms.

### SUMMARY

A series of 191 children with anaphylactoid purpura, who attended the Bristol Royal Hospital for Sick Children between 1952 and the end of 1967, has been analysed and compared with other published series.

The most important feature is the occurrence of renal involvement, which was found in 33 per cent of cases in this series. This was most likely to affect the older children, those with abdominal symptoms, and those who had relapses or recurrences of their symptoms. Renal involvement persisted for longer when it occurred more than two weeks from the onset of the illness. Persistent renal abnormality was found in from 2 per cent to 8 per cent of the total cases in this series.

Follow-up of children who have had anaphylactoid purpura is important in order to detect latent nephritis.

**Acknowledgements.** I wish to thank the staff of the Records Department of the Bristol Royal Hospital for Sick Children and the Bristol Royal Infirmary for finding the records and the Photographic Department for the photographs.

### References

Balf, C. L. (1951) Arch. Dis. Child. **26.** 20.

Bywaters, E. G. L., Isdale, I. and Kempton, J. J. (1957). Quart. J. Med. **26.** 161.

Derham, R. J. and Rogerson, M. M. (1952). Arch. Dis. Child. **27.** 139.

Derham, R. J. and Rogerson, M. M. (1956). Arch. Dis. Child. **31.** 364.

Diamond, L. K. (1955). A.M.A. J. Dis. Child. **90** 544.

Gairdner, D. (1948). Quart. J. Med. **17.** 95.

Lewis, I. C. (1955). Arch. Dis Child. **30.** 212.

Oliver, T. K. and Barnett, H. L. (1955). A.M.A.J. Dis. Child. **90.** 544.

Philpott, M. G. (1952). Arch. Dis. Child. **27.** 480.

Rubin, M. I. (1955). A.M.A.J. Dis. Child. **90.** 544.

Wedgwood, R. J. P. and Klaus, M. H. (1955). Pediatrics. **16.** 196.

## Figures and Tables

**FIG. 1. f1:**
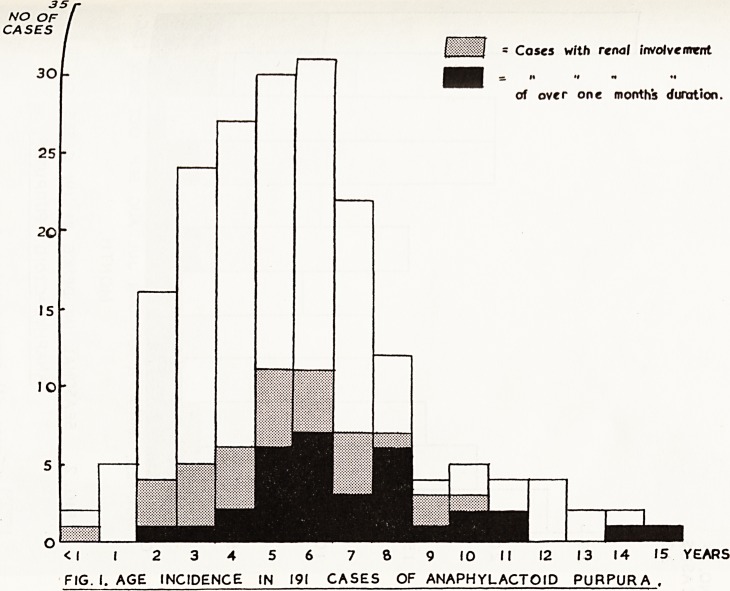


**FIG. 2 f2:**
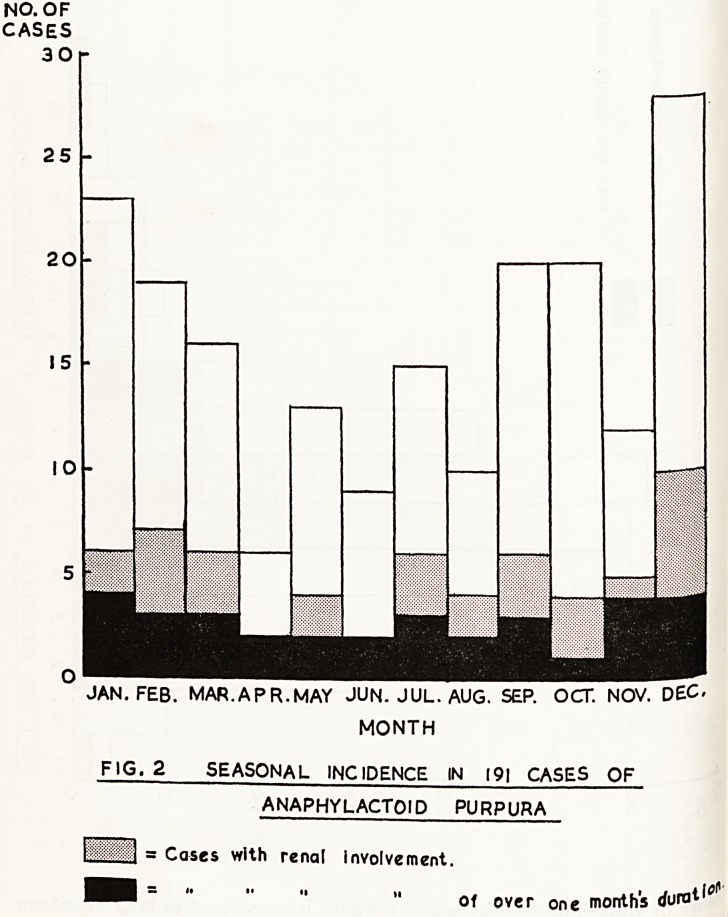


**Plate VIII f3:**
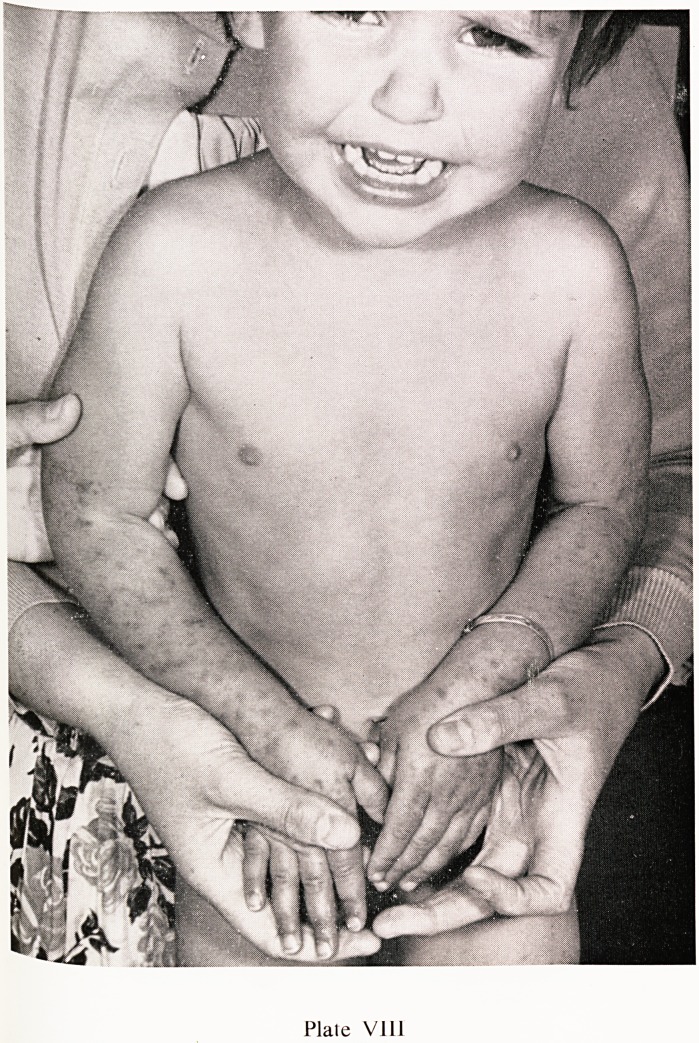


**Plate IX f4:**
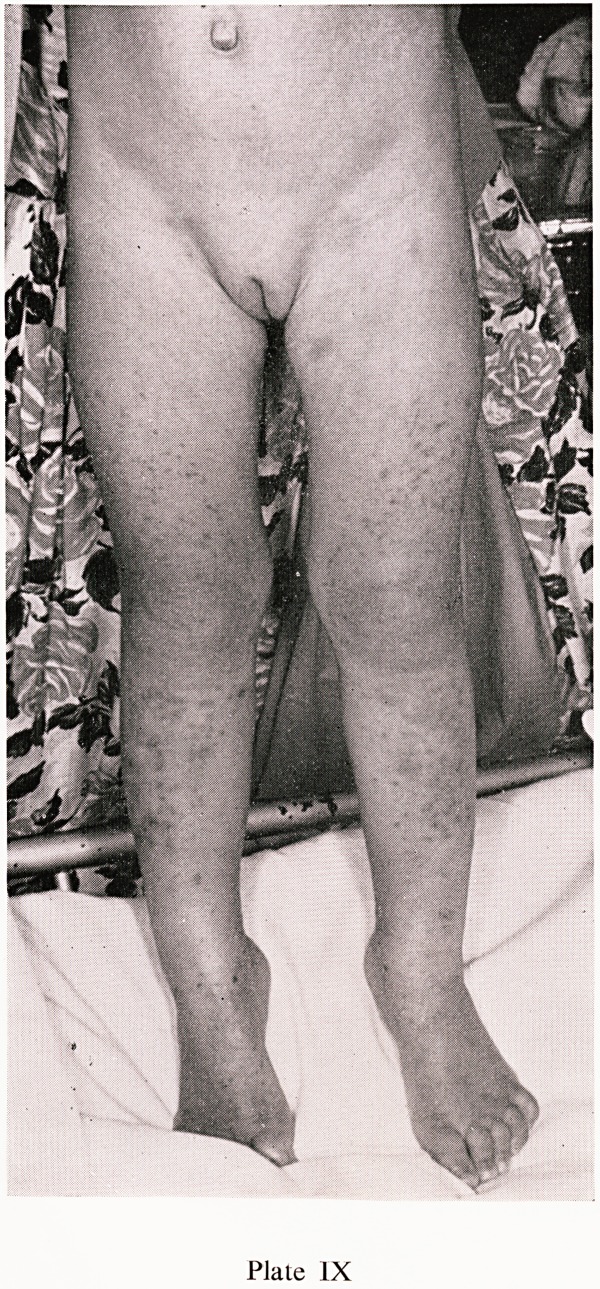


**Plate X f5:**
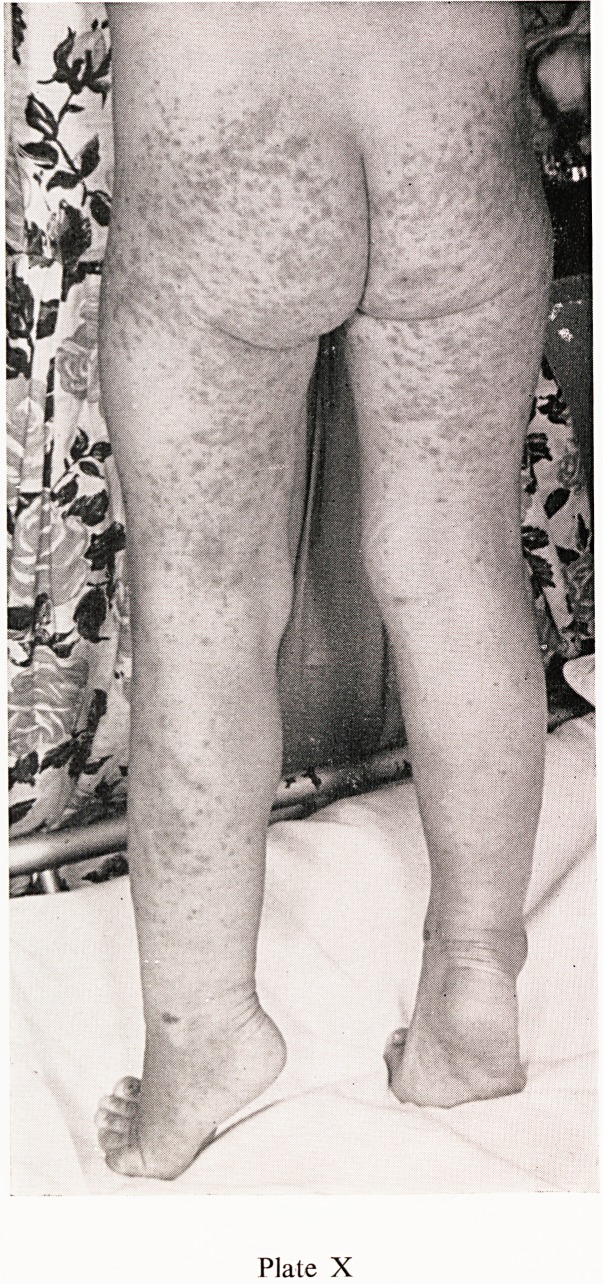


**Plate XI f6:**
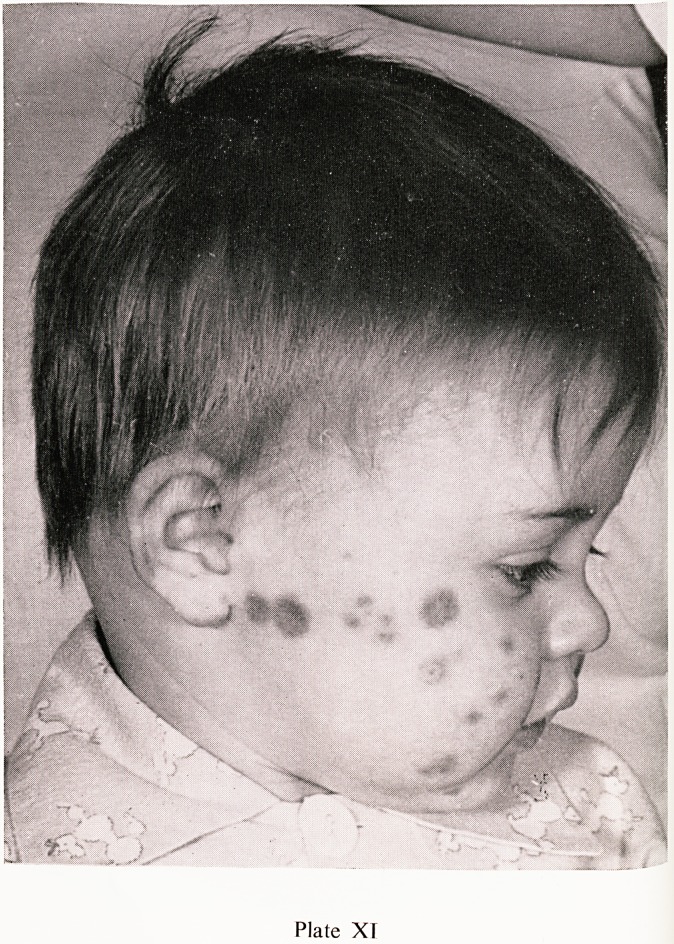


**Plate XII f7:**
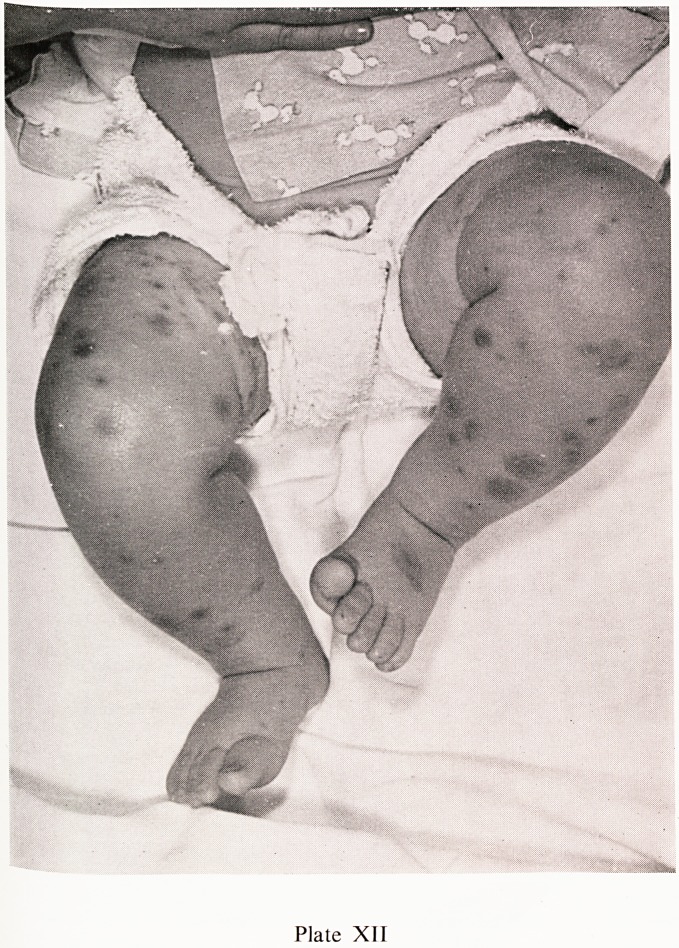


**Plate XIII f8:**